# Integration of Aqueous Two-Phase Extraction as Cell Harvest and Capture Operation in the Manufacturing Process of Monoclonal Antibodies

**DOI:** 10.3390/antib6040021

**Published:** 2017-12-01

**Authors:** Axel Schmidt, Michael Richter, Frederik Rudolph, Jochen Strube

**Affiliations:** 1 Institute for Separation and Process Technology, Clausthal University of Technology, Leibnizstraße 15, 38678 Clausthal-Zellerfeld, Germany; schmidt@itv.tu-clausthal.de; 2 Boehringer Ingelheim Pharma GmbH & Co. KG, Bioprocess + Pharma. Dev. Biologicals, Birkendorfer Strasse 65, 88397 Biberach an der Riss, Germany; michael.richter@boehringer-ingelheim.com (M.R.); frederik.rudolph@boehringer-ingelheim.com (F.R.)

**Keywords:** interfacial partitioning, cell harvest, capture, aqueous two-phase extraction, horizontal settler, static mixer, process integration, continuous phase separation

## Abstract

Substantial improvements have been made to cell culturing processes (e.g., higher product titer) in recent years by raising cell densities and optimizing cultivation time. However, this has been accompanied by an increase in product-related impurities and therefore greater challenges in subsequent clarification and capture operations. Considering the paradigm shift towards the design of continuously operating dedicated plants at smaller scales—with or without disposable technology—for treating smaller patient populations due to new indications or personalized medicine approaches, the rising need for new, innovative strategies for both clarification and capture technology becomes evident. Aqueous two-phase extraction (ATPE) is now considered to be a feasible unit operation, e.g., for the capture of monoclonal antibodies or recombinant proteins. However, most of the published work so far investigates the applicability of ATPE in antibody-manufacturing processes at the lab-scale and for the most part, only during the capture step. This work shows the integration of ATPE as a combined harvest and capture step into a downstream process. Additionally, a model is applied that allows early prediction of settler dimensions with high prediction accuracy. Finally, a reliable process development concept, which guides through the necessary steps, starting from the definition of the separation task to the final stages of integration and scale-up, is presented.

## 1. Introduction

Clarification processes and their corresponding devices, which are already well established in other industry sectors, e.g., flocculation, precipitation, or flotation, are increasingly being taken into consideration as alternatives in monoclonal antibodies (mAb) production in spite of individual limitations like process robustness, process cost, toxicity of flocculation or precipitation agents, and easy scale-up [1]. A quite promising concept is the application of aqueous two-phase extraction (ATPE) as a combined harvest and capture step, especially since this approach deals with all the aforementioned issues.

**Figure 1:**
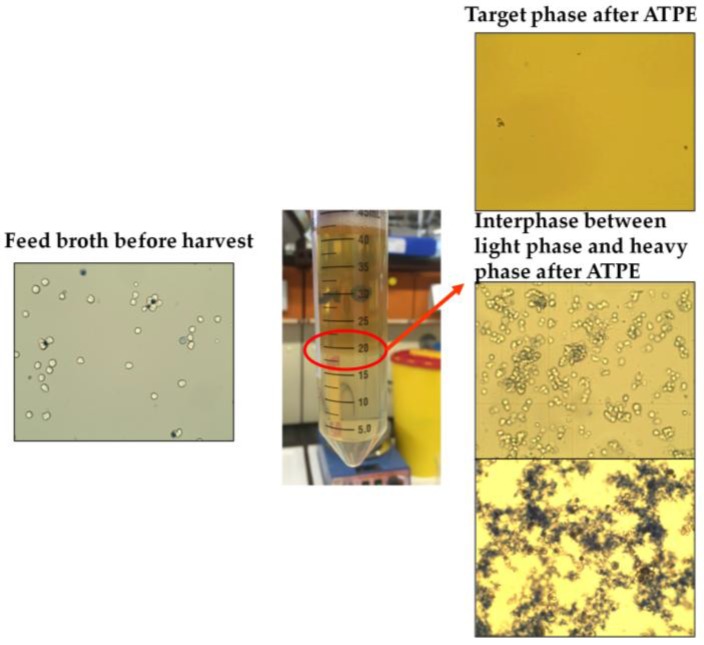
Cell clearance through interfacial partitioning. ATPE: Aqueous two-phase extraction.

Interfacial partitioning of cells, cell debris, and other bioparticles occurs when a mixed system composed of phase-forming components begins to separate into its specific light and heavy phases [2,3,4]. During the process of settling and coalescence, particles and small solid objects accumulate at the surface of the dispersed phase (Figure 1). This phenomenon can be explained by the fact that the partitioning behavior of small particles is strongly dependent on surface forces [5].

Approaches for the combination of liquid–liquid extraction (LLE) and the separation of small particles by adsorption towards a dispersed phase are well investigated. These include interfacial partitioning for the recovery of bioparticles in general, three-phase partitioning (TPP) where interfacial partitioning is combined with the precipitation of proteins, or more recently, aqueous two-phase flotation, where gas bubbles are introduced as a dispersed third phase into an already separated aqueous two-phase system (ATPS) [6,7,8]. However, most approaches so far design the process around the particle-loaded phase because the solid phase contains the product. Some research has also been conducted towards interfacial partitioning for the removal of unwanted particles from a feed stream, which is especially necessary during the clarification of cultivation broths in the manufacturing process of monoclonal antibodies. However, to establish this technology in industry, there is also the need for a process development strategy that guides through the necessary steps, starting from the definition of the exact separation task, to the experiments necessary for the determination of crucial process and model parameters, up to the design considerations for optimal equipment dimensions. In this work, an ATPE process is outlined for the clarification of up to 12,000 L of cultivation broth in a time window of less than 3 h.

## 2. Theory

### 2.1. General Considerations

Unlike in the conventional production process of mAb, centrifugation and microfiltration as harvest and clearance operations are replaced with the outlined ATPE process. After the filtration trains, the product stream can, depending on the future process strategy, be further purified by precipitation or by integrated counter-current chromatography (iCCC) for continuous capture, replacing traditional protein A chromatography, as wells as ion-exchange and hydrophobic interaction chromatography for further purification and polishing [9,10,11,12,13,14].

**Figure 2:**
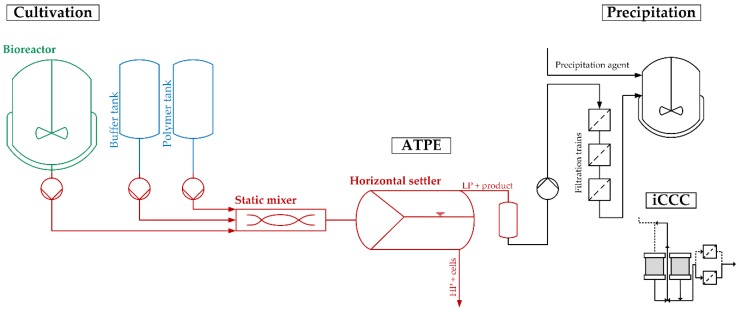
Process segment as discussed in the risk assessment. LP: Light phase. HP: Heavy phase. iCCC: integrated counter-current chromatography.

**Figure 3:**
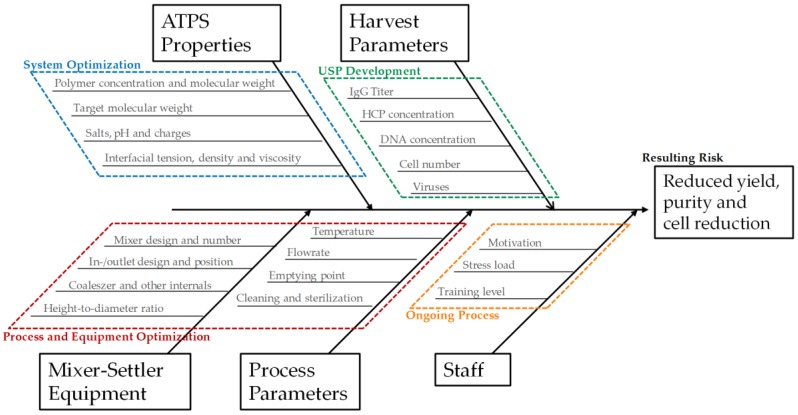
Ishikawa diagram illustrating the main groups of causes affecting yield, purity, and cell reduction in the outlined ATPE process. ATPS: separated aqueous two-phase system; HCP: host cell proteins.

The flowsheet of the discussed process, including the two purification and polishing operations, is shown in Figure 2. It provides a precise and straightforward view of how ATPE is integrated into the overall purification strategy. To quickly get an overview of all process-relevant effects, it is helpful to construct a cause-and-effect diagram, also known as an Ishikawa or fish bone diagram. It is meant to illustrate the different possible sources of reduced or insufficient process performance. The diagram is constructed of primary branches, which organize specific groups of effects. They can be fanned out further into major branches, representing major causes. Minor branches can be integrated as well to show the relationship between cause and effect in even more detail. Most diagrams have environment, people, materials, equipment, measurement systems, and methods as the primary branches. Though the diagram can be constructed like this, the causes and effects specific to the outlined ATPE targets is shown in Figure 3.

The groups of causes in the diagram are set up by considering so-called prior knowledge. The green branch summarizes the factors that arise from upstream processing (USP) during cultivation and harvesting of the target parameters in this process step. There are several factors that can be optimized during upstream process development, like cultivation time, feed media, and supplement compositions, etc. An extensive overview and methodology for USP optimization has been published by Gronemeyer et al. [15]. The overall cell number or cell concentration is very important to consider, since ATPE as a harvest operation aims to remove most of the particles to lower the burden for the subsequent filtration trains. In addition to that, higher cell numbers decrease the settling velocity inside the apparatus as during interfacial partitioning the cells adsorb on the dispersed phase and hinder the coalescence of the droplets [3,16,17]. The effect of overall protein content, which is, for the most part, the sum of the mAb and the host cell proteins (HCP), needs to be considered as well. In most cases, protein partitioning between the specific light phase and heavy phase is a function of protein concentration, where total partitioning is only observable for low protein concentrations [18,19]. However, by increasing the polarity of the heavy phase, protein solubility can be significantly lowered due to salting-out effects, resulting in very high partitioning coefficients for the target component towards the light phase [18,20,21]. There are two main side-effects of this: firstly, salting-out very often affects HCP in the same way as mAb, which means that achieving high yield comes at the cost of low protein-based purity. Secondly, total partitioning of all proteins, in combination with purposely lowering the volume of the light phase in order to concentrate the target component, can lead to protein saturation and precipitation [22,23]. 

The considered effects are also included in the second primary branch, which organizes the effects of ATPS composition and phase properties (marked blue in Figure 3). The effects of ATPS type, composition, and phase properties are discussed widely in literature [21,24,25,26,27,28]. The identification of a potential operation space should, at this point, already have occurred in a system optimization study [29].

The third primary branch organizes the influences of the applied equipment (marked red in Figure 3). There are several potential devices available for LLE in general, and ATPE specifically. An extensive overview was published by Espitia-Saloma et al. [30]. In the outlined ATPE process, the combination of a static mixing pipe and a horizontal settler is applied. Although gravity settling is a relatively robust process, even slight changes to inlet geometry or the installation of a baffle have been shown to have a noticeable impact on the settling behavior [31].

As a further step, a risk assessment considering the relative impact and the relative occurrence of the different branches is applied. This assessment considers factors like temperature, which influences the settling behavior, and changing flow rates, which influences the hydrodynamic residence time and the specific power input.

**Figure 4:**
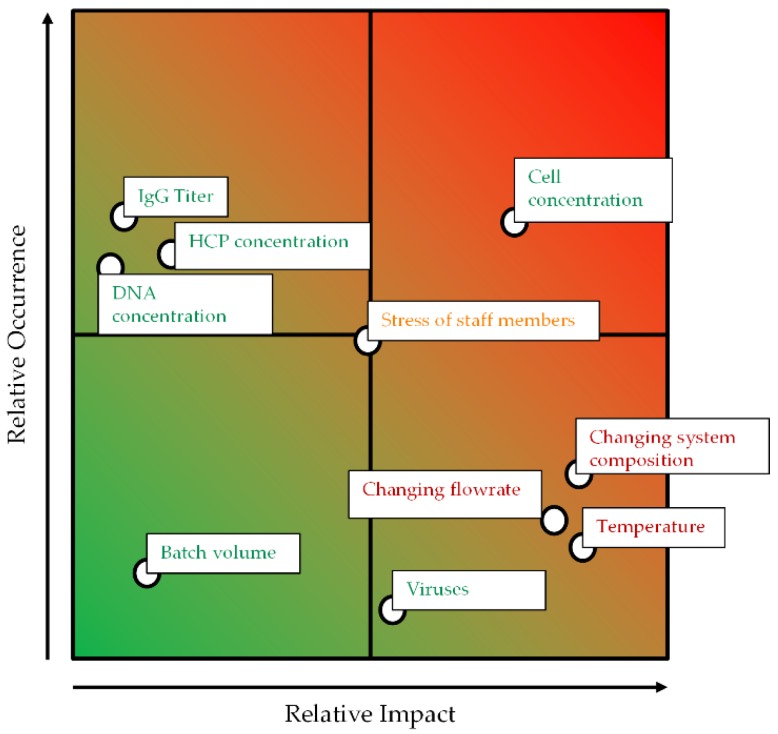
Occurrence–impact diagram for the outlined ATPE process.

Both parameters—temperature and changing flow rates—can potentially negatively impact the yield, purity, and cell reduction performance of the ATPE as illustrated in Figure 4. Increasing process temperatures can alter the intrinsic material properties of the phase-forming components which, like reducing the viscosities, could result in the creation of smaller droplets and, in the presence of cells, cell debris, and other solids, very large separation times [31]. However, coalescence kinetics in general are accelerated at higher temperatures, such that particle-free ATPS separation times can be reduced. Furthermore, the phase equilibrium itself is dependent on the temperature, since it affects the composition of the specific light and heavy phases, and therefore also changes the partitioning behavior of the target and side components [32,33,34]. The total flow rate determines the hydrodynamic residence time within the apparatus, but can result in an unnecessary firm dispersion. It is even possible to change the point of phase inversion of the system, which drastically affects the settling rates [35,36].

### 2.2. Process Development

Process development starts by defining the design space that is feasible from an economic point of view, but also safe in terms of reliable process performance. Since ATPE in this work is applied mainly as a clarification operation, the parameter which characterizes the process performance of this step, is the reduction in total cell number. One important quality measure for the monitoring of subsequent filtration, precipitation, or chromatography steps is the particle load of intermediate pools. This is because, other than the cells themselves, cell debris (precipitated material, etc.) can also lead, above certain values, to either a blockage or the underperformance of these further purification and polishing steps. Particle load can be characterized by turbidity measurements or state-of-the-art DLS (dynamic light scattering) methods. Based on so-called prior knowledge, the equipment chosen for mixing the phase-forming components is a static mixing pipe, which offers gentle and homogeneous mixing at the same time. ATPS loaded with bio-particles tends to form strong emulsions when too much power is introduced into the system [31]. The specific light and heavy phases are separated in a horizontal settler unit. This apparatus is ideal for single stage LLE since it is easy to scale-up and is proven to show reliable separation performance even when confronted with high particle burdens.

### 2.3. Batch-Settling Behavior

To enable the early prediction of settler dimensions, other than physical properties, settling and coalescence rates must also be determined.

The settling behavior of an ATPS can be mathematically described by three model parameters. The aim is to describe the influence of different material parameters on the separation behavior. The system is separated when the majority of the phases have settled and an interphase has formed.

(1) $$\frac{dh}{dt}=\frac{\sigma }{\mu_{c}}\;const.{\left(\frac{\mu_{d}}{\mu_{c}}\right)}^{a}{\left(\frac{\sigma_{w}}{\sigma }\right)}^{b}{\left(\frac{\Delta \rho }{\rho_{c}}\right)}^{c}$$

The settling time is dependent on the ratio of the interfacial tension between the phases (*σ*) and the viscosity of the continuous phase (*μ_c_*). The values are composed of the dispersed (*μ_d_*) and continuous viscosities, the interfacial tension of water (*σ_w_*) and the two phases, as well as the density difference of the dispersed and continuous phases (Δ*ρ*) and the density of the continuous phase (*ρ_c_*). The exponents *a*, *b*, and *c* are calculated numerically based on experimentally-determined settling times [37].

### 2.4. Early Prediction of Settler Dimensions

To get a rough estimation of the dimensions of the apparatus, volume flow ($\dot{V}$) is calculated, which is dependent on the entire volume (*V_total_*) of the ATPS in relation to the predetermined process duration (*t_total_*):

(2) $$\dot{V}=\frac{V_{total}}{t_{total}}$$

The length can then be calculated using volume flow and the predetermined residence time (*t_RDT_*), as well as the cross-sectional area of the device (*A_S_*):

(3) $$L_{S}=\frac{\dot{V}\times t_{RDT}}{A_{S}}$$

Further influencing variables, such as the drop size, sedimentation rates, or other parameters influencing coalescence, are not taken into account. This quick estimation is often referred to as minimum-apparatus-volume (MAV).

### 2.5. Henschke Method for Settler Dimensioning

**Figure 5:**
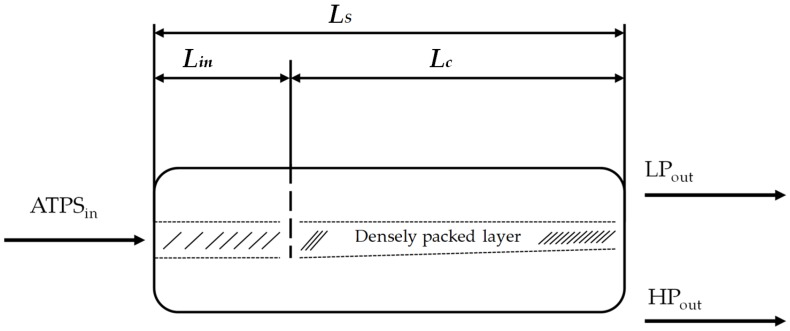
Structure of the settler unit [38]. ATPS: Aqueous two-phase system. *L_S_*: Length of the separator. *L_in_*: Inlet length. *L_c_*: Coalescence length.

A procedure to calculate the dimensions of horizontal settler units was introduced in 1994 [38]. It was derived from aqueous organic systems and is investigated here in terms of its suitability for bio-particle loaded aqueous two-phase systems. In this model, the power input, sedimentation rate, and coalescence behavior of the single droplets are taken into account. To examine these influencing factors more precisely, the length of the separator (*L_S_*) is divided into two regions: the inlet area of the dispersion (*L_in_*) and the coalescence region (*L_c_*) (Figure 5).

In the inlet area, occurring turbulences and the formation of a droplet layer are considered. Within the apparatus, drop coalescence and the formation of the densely packed layer (*h_p_*) are of greater interest. The change in the densely packed height can be calculated as follows:

(3) $$\frac{dh_{p}}{dl}=-\frac{{\dot{V}}_{Dis}\times \left(11.3\times s\times \eta_{Dis}+126\times \left(\eta_{c}+\eta_{d}\right)\right)}{h_{p}\times D_{S}^{3}\times {\bar{\varepsilon }}_{p}\times \left(1-{\bar{\varepsilon }}_{P}\right)\times \Delta \rho \times g}$$

It is dependent on the dispersion volume flow (${\dot{V}}_{Dis}$), the dispersion viscosity ($\eta_{Dis}$), a slippage parameter (*s*), the diameter of the settler (*D_S_*) and the dispersed volume fraction in the densely packed layer (${\bar{\varepsilon }}_{P}$). The settling time (*t*_s_) is normalized to level out the influence of long coalescence times at the boundary surface. The compensation parameter is (C*). With the aid of these two parameters, the coalescence length of the separator can be determined as follows:

(4) $$L_{c}=\frac{t_{S}\times {\dot{V}}_{d,0}}{D_{S}\times \sqrt[{1,3}]{\frac{H_{p}\times H_{d,0}^{0,3}}{{\left(\frac{\Phi_{32,0,settle}}{\Phi_{32,0,settler}}\right)}^{0,5}\times C^{*}}}}$$

It is dependent on the final height of dispersed phase in a batch-settling experiment (*H_d,_*_0_), the volume flow of dispersed phase (${\dot{V}}_{d,0}$) and the starting droplet diameter in a batch-settling experiment ($\Phi_{32,0,settle}$) and inside the settler ($\Phi_{32,0,settler}$). The height of the densely packed layer inside the settler (*H_p_*) should be predefined. The inlet length must also be determined. It is dependent on the velocity inside the settler (*v_S_*) the volume of the inlet (*V_in_*), the diameter of the inlet (*D_in_*), earth gravity (*g*) and the dispersion band height inside the settler ($H_{Dis}$). In this case, the mean density ($\bar{\rho }$) and the hold-up (ε_0_), which corresponds to the volume fraction of the dispersed phase in the total volume, are determined:

(5) $$L_{in}=\frac{43,7{\bar{\rho }}^{0,3}v_{S}^{0,5}D_{S}^{0,4}V_{in}^{0,5}D_{in}^{0,5}}{\Delta \rho^{0,3}{\left(\Phi_{32,0}+H_{Dis}\right)}^{0,4}{\left(1-\varepsilon_{0}\right)}^{0,2}g^{0,5}}$$

(6) $$\bar{\rho }=\varepsilon_{0}\cdot \rho_{d}+\left(1-\varepsilon_{0}\right)\cdot \rho_{c}$$

(7) $$\varepsilon_{0}=\frac{H_{d,0}}{H_{c,0}+H_{d,0}}$$

The total length of the separator (*L_S_*) is composed of the inlet length (*L_in_*) and the coalescence length (*L_c_*):

(8) $$L_{S}=L_{in}+L_{c}$$

The geometry of the dispersion layer is not further considered in the simplified model. For further explanations, reference is made to Henschke et al. [38].

## 3. Results and Discussion

### 3.1. System Selection and Batch-Settling Experiments

Based on the shaking flask results, focus is placed on system point 1 (SP1) in the following experiments, since the achievable yield is the highest of the investigated compositions at around 95%, while also showing high cell reduction capabilities. Concentration of IgG is nearly doubled in SP4, due to its phase-split, however, this comes at the cost of insufficient cell reduction (Table 1).

**Figure 6:**
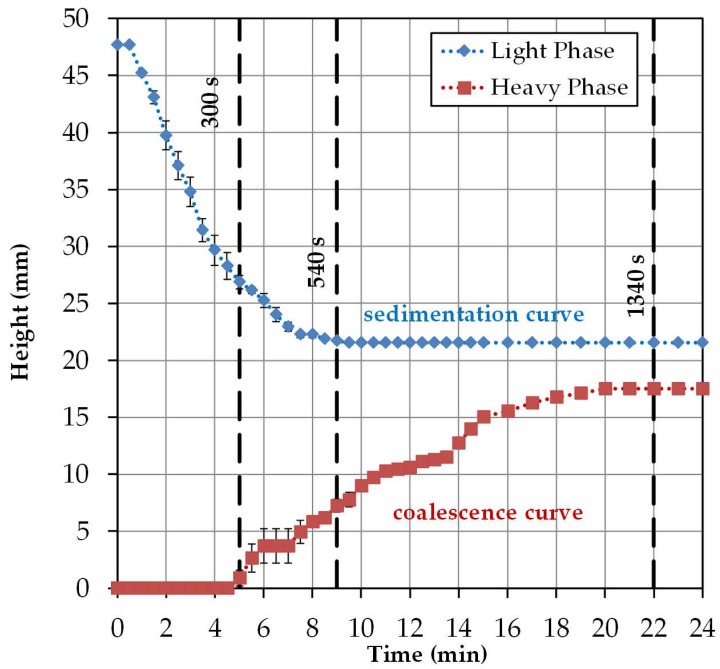
Settling curves obtained by triplicate batch-settling experiments.

In Figure 6, the height profile for the specific light and heavy phases observed in the batch-settling experiments is plotted. The specific light phase begins at a reactor height of approximately 47 mm with settling, and is complete after approximately 9 min at a reactor height of approximately 21 mm. The heavy phase begins to visibly coalesce with a delay of 5 min and has settled after about 20 min at a height of about 17 mm. This is related to the delayed coalescence of the dispersed droplets. In Table 2 the resulting sedimentation and coalescence rates, including the standard errors, are listed.

The results from the settling experiments are used below to calculate the exponents of the Mistry model. This model only considers the settling behavior of the interphase. This is divided into two areas. In the first section, up to 9 min sedimentation of the specific light phase and coalescence of the heavy phase are observable. After that, only the heavy phase coalesces. The height change of the interphase can be determined from the batch-settling experiments. The results are shown in Table 2.

The interphase, up to the time of 9 min, settles down at approximately 3.7 mm per minute. With the range up to 22 min, a sedimentation rate of 2 mm per minute is obtained. The results of the settling behavior of the interphase can be used in Equation (1) to determine the exponents *a*, *b*, and *c* (Table 3). The other parameters for the calculation are listed in Table 4.

**Figure 7:**
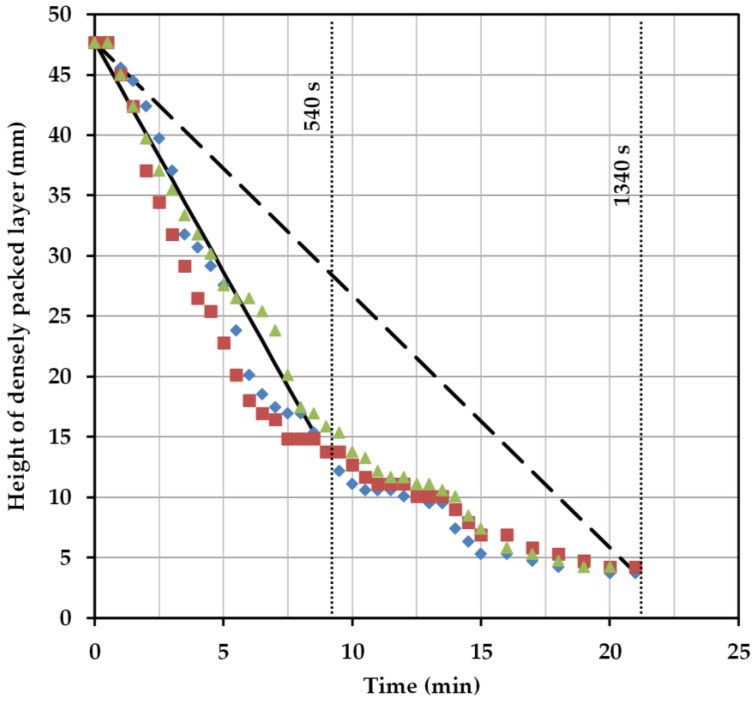
Height of the densely packed layer from triplicate batch-settling experiments (red, green, blue) as well as from the calculated separation rates (straight lines).

For 9 min, a correlation between the calculated settling time and the experimental settling time can be seen. The linear course of the interphase, which results from the deposition of both phases, is well represented by Equation (1). The exponential course of the settling curve results when the sedimentation behavior is considered up to complete separation. This results from the much longer settling time of the dispersed, heavy phase (Figure 7). The applied approach can not reflect this trend. In order to further verify the results, the settling times of other ATPS would have to be compared experimentally and theoretically. Furthermore, cell-loading and power input must be taken into account, because they influence the settling behavior of the system.

### 3.2. Model-Based Design

For the calculation of the separator length some parameters were fixed in advance. The hydrodynamic residence time for the MAV calculations equals the settling times (*t_s_*) from the batch-settling experiments. In contrast, these settling times do not equal the residence times in the more detailed calculation because this model considers not only the inlet length, but also the actual height of the densely packed layer at the apparatus end, as well as the mean droplet size. The separation times used in both calculations are 300, 540, and 1340 s.

In the calculations, the ratio *D_S_*/*L_S_* = 1/5 is chosen to achieve comparability for the MAV and the detailed calculations of the laboratory settler devices used in the experiments. The specifications for the inlet diameter (*D_in_*) and the inlet length of the separator (*L_in_*) are based on the dimensions of the laboratory separator for small volumes as well. If the system volume exceeds 160 L, the specifications are scaled-up to the dimensions of the pilot-scale device. The results of the laboratory experiments also reveal a slightly higher dispersion level (*H_Dis_*) than the densely packed dispersion layer (*H_p_*) at the end of the settler. The height of the densely packed dispersion layer is set in relation to the diameter of the settler. In addition, the height of the densely packed dispersion layer varies depending on the predetermined separation time. For this reason, a factor is used in the calculation linking the height of the densely packed dispersion layer to the diameter of the separator. This results in the following ratios:

- *H_p_*/*D_S_* = 0.54 at a cut-off time of 300 s,
- *H_p_*/*D_S_* = 0.303 at a separation time of 540 s,
- *H_p_*/*D_S_* = 0.1 with a separation time of 1340 s.

**Figure 8:**
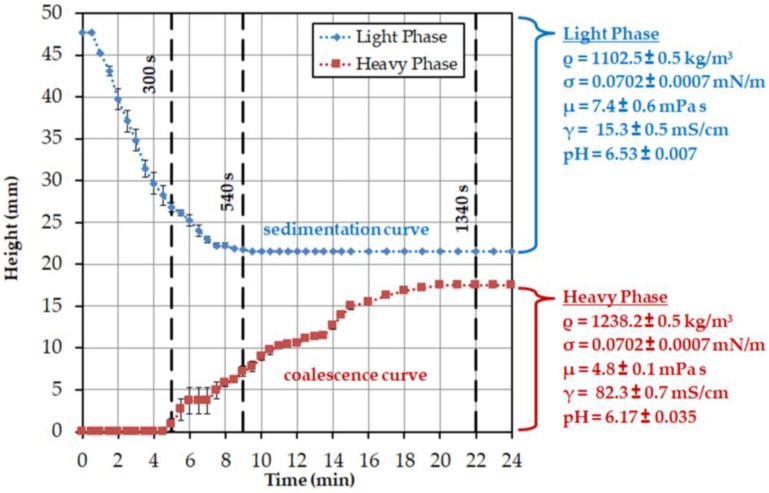
Marked separation times for the calculation from the settling experiments.

The ratio is obtained from the filling height of the batch-settling reactor and the height of the experimentally-determined densely packed layer (Figure 8).

Firstly, a cut-off time of 300 s (hereinafter called H300) is considered. At this point, the light phase has not yet completely settled. However, there is sufficient separation to retrieve clear phase at the top. The interphase is strongly pronounced at this point and it is possible that a large part of the cells are withdrawn together with the heavy phase. However, there is also the risk of losing a small amount of the specific light phase.

A separation time of 540 s is considered next (hereinafter, H540). At this point, the specific light phase in the settling test has completely settled down. The specific heavy phase can be withdrawn together with cells between the dispersed droplets without obtaining significant losses in the yield.

Complete separation of both phases is now observed at 1340 s (hereinafter called H1340). Both phases are completely separated now and it is possible to remove them separately from the separator. Removal of the densely packed layer in combination with the cells in between is only possible in this case if the phase is withdrawn very close to the phase boundary.

The length of the separator is calculated in each case for different specifications at a fixed system volume, and only for variation in the diameter of the separators to a ratio of 0.2. The investigated system volumes vary between 2 L and the industrially realizable volume of 24,000 L (roughly 12,000 L cultivation volume). As before, the systems should be separated within 3 h.

**Figure 9:**
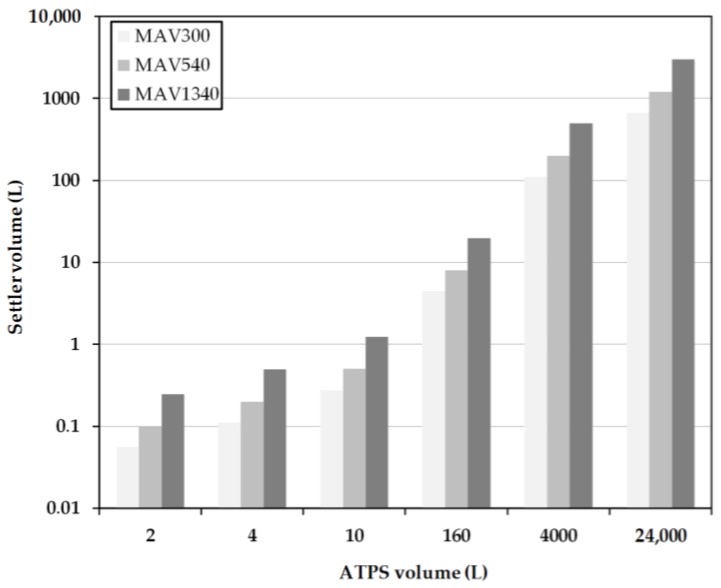
Settler volume as a function of residence time for different system scales obtained for the MAV method. MAV: minimum-apparatus-volume. MAV300, MAV540 and MAV1340 represent separation times of 300, 540 and 1340 s.

**Figure 10:**
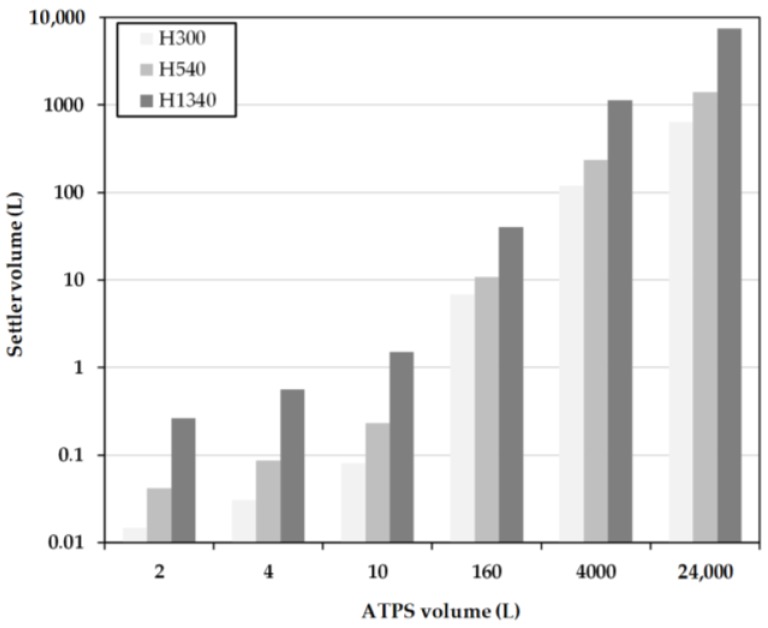
Settler volume as a function of residence time for different system scales obtained for the simplified Henschke method. H300, H540 and H1340 represent separation times of 300, 540 and 1340 s.

At low system volumes between 1 L and 10 L, the sizes of the separators according to H1340 are significantly larger than the designs according to the MAV-method (Figure 9) and H300 and H540, respectively (Figure 10). This is due to the sm all, predetermined height of the densely packed layer at the end of the separator. This results in a very large separator length, necessary to realize such a complete separation. In comparison, the settler volumes at H300 are very small due to the early withdrawal of the light phase and the accompanying larger densely packed layer. In the designs for H540, the settler dimensions are approximately 20 times smaller than the system volume. Between the increasing system volumes, a linear profile of the separator volume is indicated.

### 3.3. Process Performance

To evaluate the validity of the early prediction of settler dimensions, experimental studies were performed at lab- and pilot-scale. 

**Figure 11:**
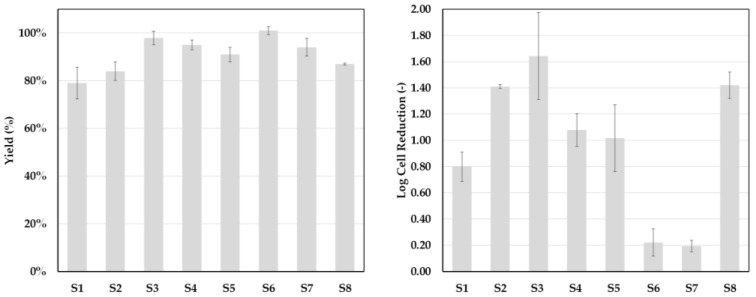
Yield and cell clearance for eight continuous settling experiments (S1–S8).

Figure 11 shows the results of eight continuous settling experiments that were conducted utilizing a settler with a nominal diameter (DN) of 50 mm (750 mL hold-up). Product yield was, across the experiments, higher than 80%. Cell reduction was at least 1 log or higher, except for experiments S6 and S7. The reason for this was a change in flowrate. S6 and S7 were part of a phase inversion study. By increasing the volume flow, effects on yield, phase separation, and cell clearance should be determined. The relatively high yields of 90–100% indicate that mass transfer, as well as phase separation, is not negatively affected by the increase in flowrate.

**Figure 12:**
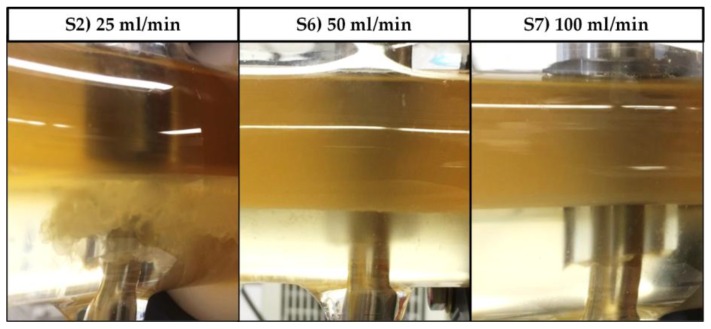
Exit area of the DN50 settler at three different flowrates but an otherwise identical set-up (DN: Diameter nominal). In experiment S2, cells can be retrieved together with heavy phase through bottom phase exiting (left).

**Figure 13:**
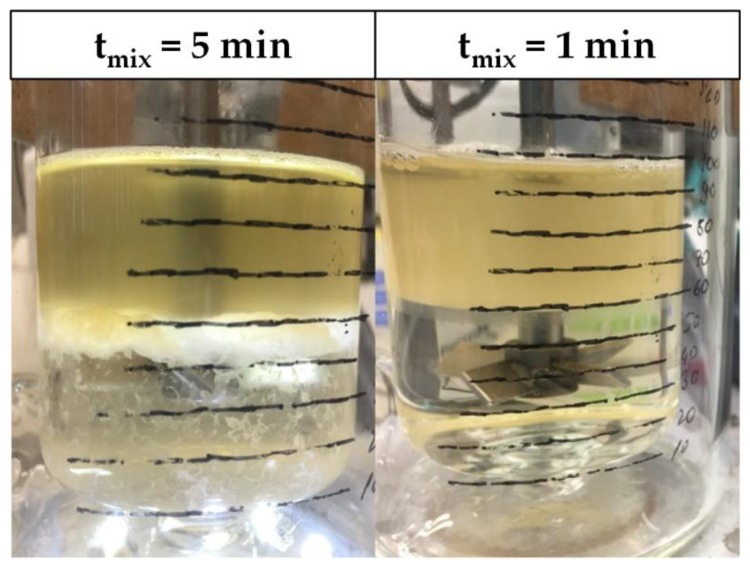
SP1 after batch-settling. Clear light phase due to interfacial cell partitioning (**left**). High turbidity in light phase, because of insufficient mixing (**right**).

However, as can be seen in Figure 12, since the length of the static mixing pipe is fixed, the residence times in S6 and S7 are decreased not only in the settler, but more importantly in the mixing pipe as well. As a result, the phase-forming components are insufficiently mixed and no interfacial partitioning occurs. This system behavior can be reproduced in the batch-settling experiments as well. Leaving the standard set-up conditions constant, but reducing the mixing time from 5 to less than 1 min, results in an absence of interfacial partitioning (Figure 13). 

### 3.4. Comparison of Experiment and Model

**Figure 14:**
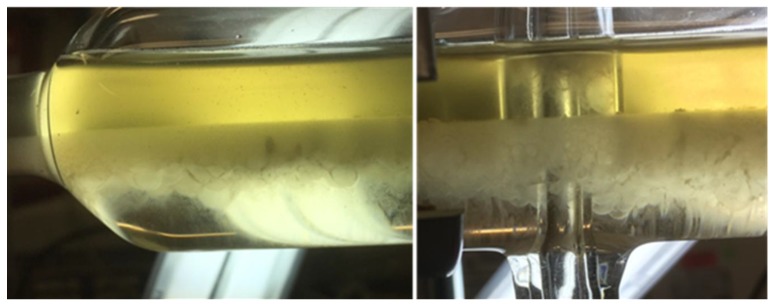
Side view of the laboratory settler (DN35, 175 mL hold-up. DN: diameter nominal) during continuous settling operation (300 s residence time). The image shows the inlet area (**left**) and the heavy phase outlet (**right**).

In Figure 14, the phase separation profile inside a laboratory settler during continuous settling operation is shown. A volume flow of 35 mL/min is constantly separated during the procedure. For 3 h process time, this results in a processed ATPS volume of around 6.3 L. Complete phase separation of light and heavy phases is achieved.

Also, the pronounced interphase, composed of cells and densely packed dispersed phase, meets the coalescence profile from the batch-settling experiment (Figure 8). The continuous settling experiments executed in the DN50 settler, except for S6 and S7 for the above-discussed reasons, show very narrow interphase bands (Figure 12, left), which is also in accordance with the batch-settling profile, considering that the residence times are beyond the 1340 s marking line.

**Figure 15:**
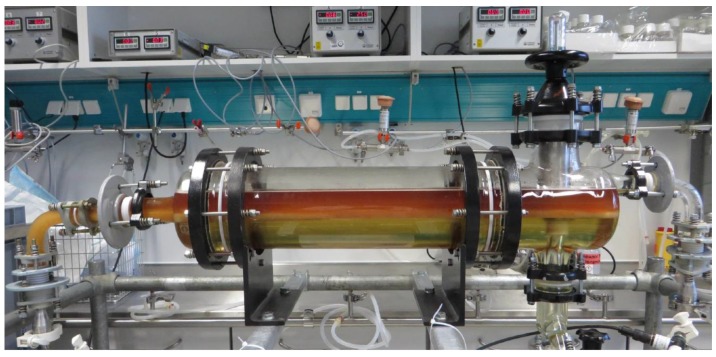
Side view of the pilot-scale settler (DN150, 12.6 L hold-up) during continuous settling operation (900 s residence time).

Finally, a pilot-scale operation of the continuous settling process is discussed. Figure 15 shows the experimental set-up. Like in the lab-scale studies, phase-forming components, after passing through a static mixer pipe, continuously enter the apparatus. To ensure total sedimentation of the light phase, a residence time of 900 s was adjusted. Due to a small measurement inaccuracy of the flow meters, the PEG content in the system was lower (13 instead of 15.5 w%) and the percentage of phosphate rose from 15.5 to 18.7 w%. Thus, the operating point is closer to SP4 than to SP1. This results in phase inversion, which is also visible in Figure 15. In order to obtain a predominantly light phase for the filtration train, large parts of the light phase with the cells were withdrawn through the immersion tube during operation, which led to a product yield of 80.4%. For better comparability, the same process was carried out in batch-settling mode simultaneously. The comparisons in yield, cell reduction, and subsequent filterability are listed in Table 5.

Both operation modes clarified approximately 160 L total volume within the aimed-at time-frame of less than 3 h. No filter blockage occurred, however, due to the discussed shift in system composition, the product phase from the continuous settling process showed a higher yield loss. Also, the necessary filtration pressure was significantly higher.

**Figure 16:**
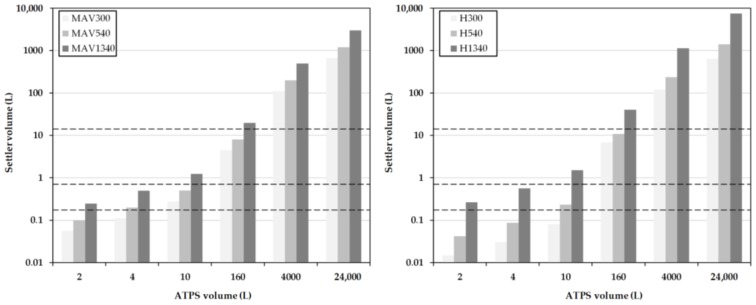
Settler volume as a function of residence time for different system scales obtained for the MAV (**left**) and simplified Henschke method (**right**). The dashed lines indicate the experimentally-tested scenarios (from top to bottom): 12.6 L, 0.75 L, and 0.175 L settler volumes for 160 L, 10 L, and 4 L ATPS volumes.

Compared to the settler dimensions, modeled only on the basis of batch-settling experiments (Figure 16, dashed lines indicate tested scenarios), the used DN150 settler (12.6 L hold-up), is very close to the calculated 10.9 L settler volume (H540), and in accordance to the thick dispersion layer significantly smaller than 40 L (H1340), which would be necessary for a narrow dispersion band.

## 4. Materials and Methods

### 4.1. Aqueous Two-Phase Systems

In this work, four ATPS are considered. The components of the individual systems with their approximate phase splits are listed in Table 6.

### 4.2. Cultivation

The fed-batch cultivation of Chinese hamster ovary cells (cell line CHO DG44), used for mAb production, is carried out in a commercial serum-free medium. The cells were cultivated one week at 37 °C, 5% CO_2_, and 130 rpm in shaking flasks. 

### 4.3. Analytical Procedure

Protein A chromatography (PA ID Sensor Cartridge, Applied Biosystems, Bedford, MA, USA) was carried out for the determination of IgG concentrations. The buffers were PBS A (pH 7.4) for binding and PBS B (pH 2.6) for elution. For the analysis, a flow of 1.6 mL/min was applied. The injection mass for the calibration varied between 10 μg and 95 μg. Sample quantities of 50 μg are applied. The measured signals (280 nm) were evaluated via peak areas.

For size exclusion chromatography (SEC), a 1 M disodium hydrogen/sodium dihydrogen phosphate buffer containing 1 M sodium sulfate was used (Merck KGaA, Darmstadt, Germany). The column was a Yarra™ 3 μm SEC-3000 (Phenomenex Ltd., Aschaffenburg, Germany). The analysis was carried out at a flow rate of 0.35 mL/min and a duration of 23 min.

Cells are counted using a Motic BA 310 microscope (Motic Deutschland GmbH, Wetzlar, Germany). A sample is taken and dyed with trypan blue ((Sigma-Aldrich, St. Louis, MO, USA) in order to screen dead cells. The cells are applied to a Neubauer counting chamber (Brandt, 0.1 mm depth and 0.0025 mm^2^).

Density (DMA 500, Anton Paar GmbH, Graz, Austria), pH (InoLab pH 720, WTW, Weilheim, Germany), and conductivity (InoLab pH 720, WTW, Weilheim, Germany) were measured for each ATPS. Dynamic viscosity (Rotary viscometer of the type HAAKE™ ViscoTester™ 550, ThermoFisher Scientific™, Waltham, MA, USA) and interfacial tension (Spinning Drop Tensiometer (SDT), Krüss GmbH, Hamburg, Germany) were determined for model calculations as well.

### 4.4. Procedures

#### 4.4.1. Shaking Flask Experiments

The system was weighed in tubes and was sealed. PEG 400 is added first and afterwards the broth and buffer must be added quickly. The samples rest for at least 15 min. A sample, approx. 8–10 mL, was taken from the light phase and heavy phase and centrifuged in 15 mL tubes at 3000 rpm for 10 min to separate phase residues and solids from the product.

#### 4.4.2. Batch-Settling Experiments

At the beginning of the experiment, the cell count in the cultivation flask was determined. The cell count was adjusted via dilution with a cell free broth or a concentration of the cells using a centrifuge. The predetermined stirrer speed of 300 rpm was set up with a tachometer in order to obtain a defined power input. Furthermore, 100 g of ATPS was weighed into the reactor at a cell number of around 200E5 cells/mL and then mixed for 5 min with a blade stirrer (4 blades, 45° blade angle, 29 mm diameter, 5 mm height). These parameters were sufficient for mixing the system. The settling height of the light phase and the heavy phase was recorded in 30 s intervals. From a settling time of 15 min, the values were recorded every minute. After separation of the light phase, heavy phase, and interphase, a sample of each phase was taken. The samples were centrifuged at 3000 rpm for 10 min to separate cell and phase residues.

#### 4.4.3. Continuous-Settling Experiments

In order to prevent sedimentation of the cells in the feed vessel, the feed vessel was stirred on a magnetic stirring plate at about 290 rpm. The components were combined in two Y-pieces by three peristaltic pumps (Ismatec IP65, Cole-Parmer GmbH, Wertheim, Germany) according to mass-flow measurements (Mini Cori-Flow, Bronkhorst Deutschland Nord GmbH, Kamen, Germany) and mixed by static helix mixers (ESSKA.de GmbH, Hamburg, Germany). The mass-flow was recorded during the test by a Labbox (Labbox 3 M, HiTec Zang GmbH, Herzogenrath, Germany). At the outlet of the separator, the phases were collected separately from each other. During the experiment, samples were taken in order to detect changes in product yield or cell reduction over time.

## 5. Conclusions

**Figure 17:**
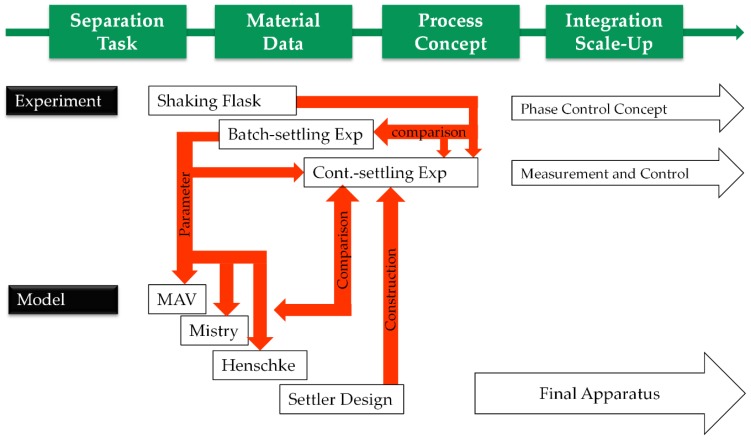
Process development strategy.

The presented work displays development and implementation strategies for aqueous two-phase extraction as a cell harvest operation (Figure 17). After defining the operation space for the specific separation task in terms of cell reduction, yield, and process time by a limited number of shaking flask experiments to obtain equilibrium composition and material data by lab-scale batch and continuous settling tests, early predictions of necessary settler dimensions are possible.

The batch-settling experiments in particular enable the identification of the separation times that are necessary to obtain the desired sedimentation profile inside the settler. Knowledge about the sedimentation rates can then be used for more sophisticated calculation of settler dimensions. For 3 h continuous processing of up to 12,000 L cultivation broth, a settler with a length of 3.6 m and a volume of approximately 1.4 m^3^ is proposed (Figure 16, H540).

The investigated operating point is characterized by a yield of between 80 and close to 100%, as well as up to 2 log step reductions in cell number. For small system volumes (2–10 L), the calculated settler dimensions are consistent with laboratory tests as well as with pilot-scale volumes of up to 160 L cultivation broth. In order to achieve the above-described yield, the correct system composition (SP1) must be observed. In the case of deviations, this may lead to a reversed direction of phase inversion. Since the heavy phase must be dispersed for a reduction of cells, this is of upmost importance. The focus of further research is shown in an illustrated concept (Figure 17). Further work packages should optimize process integration (i.e., automation, control, and control strategy) as well as the robustness of the process. The applicability for alternative ATPS, i.e., polymer-citrate, as well as for other products, i.e., virus-like particles or different proteins, should also be investigated to evaluate the robustness of the process and the reliability of the development concept.

## Figures and Tables

**Table 1:** Yield, purity, and cell reduction of the investigated ATPS.

System Point	Yield (%)	SEC-Purity (%)	Log Cell Reduction (−)
SP1	95 ± 2.33	80.93 ± 0.02	2.08 ± 0.1
SP2	80 ± 0.39	81.97 ± 1.7	2.47 ± 0.02
SP3	63 ± 3.00	67.98 ± 1.8	3.45 ± 0.2
SP4	80 ± 1.62	80.98 ± 0.03	0.61 ± 0.02

ATPS: aqueous two-phase system; SEC: size exclusion chromatography; SP: system point.

**Table 2:** Sedimentation and coalescence rates in mm/min obtained by triplicate batch-settling experiments.

Time Interval	d*h*_p_/d*t* (mm/min)		
	1.1	1.2	1.3
540 s	3.8	3.87	3.62
1340 s	2.09	2.07	2.17

**Table 3:** Parameter set for the calculation of phase separation rates.

Time Interval	*a*	*b*	*c*
540 s	0.6230	0.1604	3.1096
1340 s	0.6276	0.0824	3.1644

**Table 4:** Material data for the calculation of phase separation rate.

*σ* (mN/m)	*σ*_w_ (mN/m)	ρ (kg/m^3^)	Δρ (kg/m^3^)	$\eta_{c}$ (Pa⋅s)	$\eta_{d}$ (Pa⋅s)
0.0702	72.75	1102.5	0.1337	0.0074	0.0048

**Table 5:** Comparison of batch and continuous ATPE of 160 L system volume. The pressure increase refers to 117 L/m^2^ area-specific filtration volumes.

Mode	Yield IgG (%)	Log Cell Reduction (−)	Pressure Increase (bar)
Batch	95	1.29	0.1
Continuous	80.4	0.44	0.9

**Table 6:** Composition of the investigated ATPS.

	SP1	SP2	SP3	SP4
PEG 400 (w%)	15.5	17	20	9
Buffer (w%)	40	43.75	45	53
Broth (w%)	44.5	39.75	35	38
Volume split LP/HP	1:1	1:1	1:1	1:4
